# The Tuberculosis Congress

**Published:** 1906-11

**Authors:** 


### EDITORIAL DEPARTMENT.

### THE TUBERCULOSIS CONGRESS.

As previously announced, the fourth biennial session of the American International Congress on Tuberculosis will be held in New York City November 14, 15, and 16th (inst.). The preliminary program was received too late for publication in this issue. I had expected to publish it. I am advised that the program in detail is very full and complete. Many strong and important papers have been contributed, and addresses will be made by notable men. The headquarters will be at the New Astor Hotel, where there will be a committee who will give delegates all desired information, including the special rates secured at hotels and boarding houses, and programs and badges will be then distributed. The fare over all routes will be one full fare going, and one-third fare returning, good for any length of stay in New York. Delegates should get from the local ticket agent a certificate that one full fare has been paid. This certificate endorsed by the secretary will entitle the holder to one-third rate returning. Through-sleeper tickets can be secured, changing sleepers at St. Louis, via the Wabash to New York, if that route is chosen.

The management are advised that there will be a large attendance, not only from the States of North, South, and Central America, but from foreign countries in response to the invitations sent out by Hon. Elihu Root, Secretary of State. There will be a dinner on the evening of the 15th, at which it is expected ladies will be present. Of course, there will be toasts and speeches, "a feast of reason and a flow of"—"spirits." Tickets for the dinner must be spoken for in advance and paid for,—so much,—I do not know how much, —per plate. This dinner is given by the Medico-Legal Society in honor of the Congress on Tuberculosis.

It is expected that much good will result from this meeting. The discussions will take a wide range, but the keynote will be Preventive Legislation; the prophylaxis of consumption. The government must come to the relief of the people. They are being awakened to the enormity of the losses by this preventable disease, and the medical profession having done its full share in pointing out the cause, mode of dissemination and how it may be controlled, the people must now demand protective legislation at the hands of their representatives. The main plea will be for a Department of Public Health with a minister or commissioner in the cabinet of the President.

It is expected that there will be a full representation from Texas. Governor Lanham has appointed a list of fifty representative physicians of the State Medical Association, and a very large majority of them have notified Secretary Smith of their intentions to be present. Dr. Geo. R. Tabor, our popular State Health Officer, has been elected first vice-president, vice Kellogg, unavoidably absent, and is also chairman of Committee on State Sanatoria. Dr. Tabor has signified his intention of being present.

Texas is signally honored in that the principal officers are Texans, and she must keep her corner up, and lead in this great matter of sanitary reform. The government must act and at once, to stay the fearful losses by consumption.

